# Real-World Clinical Profile and Safety of Nintedanib in Systemic Sclerosis-Associated Interstitial Lung Disease: A Subgroup Analysis of Interstitial Lung Disease Data From an Interstitial Lung Disease (ILD) Specialty Clinic in India

**DOI:** 10.7759/cureus.65579

**Published:** 2024-07-28

**Authors:** Ajoy K Behera, Pratibha Sharma, Ranganath TG, Vikas Kumar, Saroj K Pati, Kulshreshth Sinha

**Affiliations:** 1 Pulmonary Medicine, All India Institute of Medical Sciences, Raipur, Raipur, IND; 2 Microbiology, Shri Balaji Institute of Medical Science, Raipur, IND; 3 Radiodiagnosis, All India Institute of Medical Sciences, Raipur, Raipur, IND

**Keywords:** connective tissue disease-associated interstitial lung disease, lung function test, nintedanib, cyclophosphamide pulse, mycophenolate mofetil (mmf), diffuse systemic sclerosis

## Abstract

Introduction: Systemic sclerosis (SSc) is a multisystem autoimmune disorder characterized by dysregulated innate and adaptive immunity. Interstitial lung disease (ILD) is a common and serious complication of SSc, often leading to significant morbidity and mortality. Consistent demographic characteristics that aid in the early diagnosis of ILD in SSc are lacking. This study aims to identify clinical and demographic parameters associated with ILD in SSc patients and assess the safety and tolerability of nintedanib with other immunosuppressants.

Materials and methods: This study is a subgroup analysis of data from the ILD clinic at All India Institute of Medical Sciences Raipur, collected between January 2022 and January 2024. We assessed the clinical and demographic profiles, high-resolution computed tomography thorax patterns, autoantibody profiles, lung function, and treatments used in the patients.

Results: We enrolled 57 patients with SSc-associated ILD. The mean age of the participants was 39.0 ± 11.1 years, with 53 (92.9%) being women. The mean body mass index was 20.4 ± 4.32 kg/m². Dyspnea was the most common symptom, followed by skin tightening and cough. Antinuclear antibody tests were positive in 92.9% of patients, and anti-Scl-70 antibodies were positive in 57.9%. Rheumatoid arthritis-SSc overlap was observed in 15.8% of patients. The mean predicted forced vital capacity was 46.5 ± 19.9%, the mean predicted total lung capacity was 64.5 ± 20.4%, and the mean predicted diffusing capacity for carbon monoxide was 46.2 ± 15.7%. The mean six-minute walk distance was 360.3 ± 81.2 meters, and the mean King’s Brief Interstitial Lung Disease score was 63.9 ± 10.7. Common radiological abnormalities included ground-glass opacities in 57.8%, traction bronchiectasis in 43.8%, and honeycombing in 28.07%. The predominant ILD pattern was nonspecific interstitial pneumonia. Patients received a combination of prednisolone (5 mg/day) with mycophenolate mofetil (63.2%), hydroxychloroquine (17.5%), cyclophosphamide (12.3%), and methotrexate (7.02%). Nintedanib, the only antifibrotic used, was administered to 17 (29.8%) patients.

Conclusions: ILD is relatively common in SSc, particularly in patients with diffuse cutaneous SSc and those with anti-topoisomerase antibodies. Female patients comprised the predominant population in this study. Patients tolerated mycophenolate mofetil and cyclophosphamide well. Nintedanib was the only antifibrotic used, and all patients tolerated the combination of antifibrotics and immunosuppressants well. Early diagnosis is crucial to slow disease progression and preserve lung function. Our results highlight the need for vigilant screening in high-risk groups and suggest that MMF, cyclophosphamide, and nintedanib can be safely incorporated into treatment regimens, offering a potential strategy to improve patient outcomes.

## Introduction

Systemic sclerosis (SSc) is a multisystem autoimmune disorder with an unknown etiology. It is characterized by dysregulated innate and adaptive immunity, repeated endothelial injury, and fibroblast recruitment, leading to extracellular matrix accumulation, tissue injury, and fibrosis [1]. Interstitial lung disease (ILD) affects 35% to 52% of SSc patients and is the leading cause of death in SSc, accounting for 20% to 40% of mortality [2,3]. Persistent symptoms of chronic cough and exertional dyspnea often indicate the onset of ILD. A high-resolution computed tomography (HRCT) scan of the thorax is a sensitive tool for diagnosing ILD, supported by lung function tests like spirometry and diffusing capacity for carbon monoxide (DLCO). Declining forced vital capacity (FVC) and DLCO suggest ILD progression [4,5]. Treatment options include cyclophosphamide, mycophenolate mofetil, rituximab, and hematopoietic stem cell transplantation [6]. Nintedanib and pirfenidone have also been used in SSc-associated pulmonary fibrosis ILD with variable success [7,8]. Common risk factors for developing or progressing ILD include male sex, diffuse cutaneous SSc (dcSSc), topoisomerase-I/anti-Scl-70 antibodies, African American ethnicity, a high modified Rodnan score, and cardiac involvement [9]. However, data on clinical and demographic parameters consistently associated with ILD occurrence in SSc are lacking, which is crucial for early identification and treatment to improve outcomes. This study aims to identify the clinical and demographic parameters associated with ILD. The secondary objective is to assess the safety and tolerability of nintedanib when combined with other immunosuppressants.

## Materials and methods

We conducted a prospective study in the ILD Clinic in the Department of Pulmonary Medicine at All India Institute of Medical Sciences (AIIMS) Raipur. The Institute Ethics Committee of AIIMS Raipur approved the study as part of the Indian Council of Medical Research Indian Network of Pulmonary Fibrosis multicenter study (approval letter no. AIIMSRPR/IEC/2020/551). This subgroup analysis includes data from our center, collected from January 2022 to January 2024. Patients with a confirmed diagnosis of SSc with chronic respiratory symptoms and HRCT thorax features of ILD were referred to the clinic. We enrolled these patients after applying the inclusion and exclusion criteria. The study included patients diagnosed with SSc referred for evaluation of unexplained dyspnea, those with evidence of ILD on HRCT of the thorax performed within the last three months, and those willing to participate. We excluded patients diagnosed with chronic obstructive pulmonary disease, asthma, or post-tubercular sequelae; those unable to perform spirometry and other lung functions; those with contraindications for spirometry (such as hemoptysis, pneumothorax, recent abdominal surgery) [10]; those with contraindications for the six-minute walk distance (6MWD) test [11]; and those unwilling to participate.

**Figure 1:**
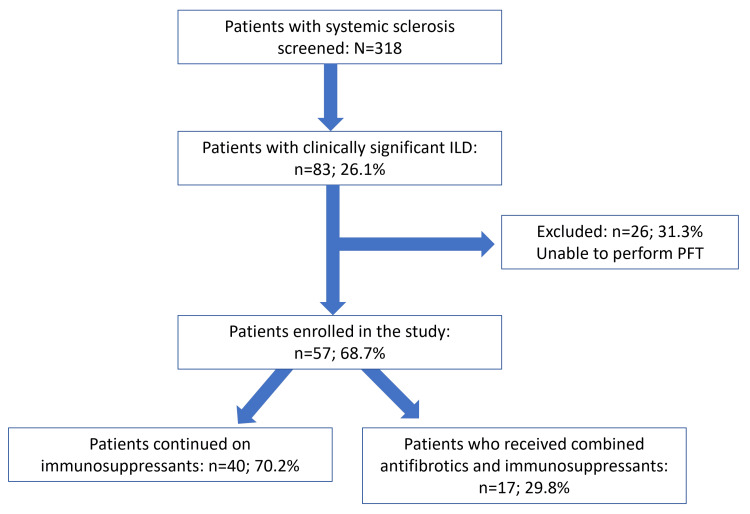
Patient flow in the study protocol PFT: Pulmonary function test including spirometry, DLCO, and six-minute walk test; DLCO: diffusing capacity for carbon monoxide; ILD: interstitial lung disease

After obtaining informed consent, we conducted clinical examinations and used the King’s Brief Interstitial Lung Disease (KBILD) questionnaire to assess patients’ quality of life [12]. We collected data on demographic profiles, serological tests, and the use of immunosuppressants. These patients underwent pulmonary function tests using the PowerCube Body+ (with Diffusion+) system (GANSHORN Medizin Electronic, Germany) as per the American Thoracic Society (ATS)/European Respiratory Society (ERS) recommendations while seated and wearing a nasal clip [10]. According to the 2005 ATS/ERS recommendations, we determined lung volumes such as residual volume, functional residual capacity, and total lung capacity (TLC) by whole-body plethysmography [13]. We conducted the 6MWD test following standard ATS recommendations [11]. Patients began nintedanib treatment after the initial assessment as required. Figure 1 shows the flow of patient selection in the study.

Statistical analysis

We analyzed the data using IBM SPSS Statistics for Windows, Version 20 (Released 2011; IBM Corp., Armonk, New York, United States). Quantitative variables were expressed as means and standard deviations. Categorical data were expressed as percentages. We used the Chi-square test to analyze categorical data and an unpaired t-test to compare the means of two unrelated groups. A p-value ≤ 0.05 was considered statistically significant.

## Results

Clinical and demographic profile

We enrolled 57 patients with SSc-associated ILD (SSc-ILD) in the study. The mean age of the patients was 39.0 ± 11.1 years. Of these patients, 53 (92.9%) were female, and four (7.1%) were male. The mean body mass index was 20.4 ± 4.32 kg/m². Most patients were non-smokers (n=56, 98.2%). The characteristics of symptoms are detailed in Table 1. Dyspnea was the most common symptom, followed by skin tightening and cough, with photosensitivity being the least common.

**Table 1:** Symptomatic profile of SSc ILD patients in the study (n=57) SSc: Systemic sclerosis; ILD: interstitial lung disease; GERD: gastroesophageal reflux disease.

Symptoms	N (%)
Dyspnea	48 (84.2%)
Skin tightness	40 (70.2%)
Cough	34 (59.6%)
Raynaud phenomenon	32 (56.2%)
Joint pain	30 (52.6%)
GERD	27 (47.4%)
Pitting of nails	24 (42.1%)
Chest pain	21 (36.8%)
Dry mouth/eyes	19 (33.3%)
Proximal muscle weakness	18 (31.5%)
Recurrent oral ulcers	16 (28.07%)
Photosensitivity	3 (5.2%)

Autoantibody profile

Antinuclear antibody was positive in 92.9% of patients. Anti-Scl70 antibodies were positive in 57.9% of patients, and rheumatoid arthritis-SSc overlap was observed in 15.8%. The mean 6MWD for anti-Scl70 positive patients was 380.1 ± 65.2 meters; for anti-Scl70 negative patients, it was 333.0 ± 93.5 meters, the difference was statistically significant (p=0.02). There were no significant differences between the anti-Scl70 positive and negative groups in demographic features, lung function, and KBILD scores.

Lung function and radiology

Most patients exhibited moderate restriction and a moderate reduction in DLCO. The mean predicted FVC was 46.5 ± 19.9%. The mean predicted TLC was 64.5 ± 20.4%. The mean predicted DLCO was 46.2 ± 15.7%. The mean 6MWD was 360.3 ± 81.2 meters. The mean KBILD score was 63.9 ± 10.7. All patients underwent HRCT of the thorax. The most common abnormalities were ground-glass opacities, seen in 57.8% of patients, traction bronchiectasis in 43.8%, and honeycombing in 28.07%. Nonspecific interstitial pneumonia was the predominant ILD pattern.

Background immunosuppression and antifibrotics

All patients received a combination of immunosuppressive therapies at the time of evaluation. They received prednisolone 5 mg/day in combination with mycophenolate mofetil (63.2%), hydroxychloroquine (17.5%), cyclophosphamide (12.3%), and methotrexate (7.02%). Serological, radiological, and treatment characteristics are detailed in Table 2. Patients with more than 10% lung involvement according to HRCT thorax, as assessed by visual estimation, were started on antifibrotic therapy. Nintedanib was the only antifibrotic used in these patients. Table 3 shows the clinical and demographic differences between patients who were started on nintedanib and those who continued background immunosuppression alone. Patients on combined therapy had lower TLC, lower DLCO, and poorer KBILD scores than those on immunosuppressants alone (p≤0.05).

**Table 2:** Serologic profile, radiological pattern, and treatment profile of SSc ILD patients SSc: Systemic sclerosis; ILD: interstitial lung disease; ANA: antinuclear antibody; RA: rheumatoid arthritis; HRCT: high-resolution computed tomography.

Characteristics		N (%)
ANA positivity		53 (92.9%)
RA–SSc overlap		9 (15.8%)
Anti-Scl-70 positivity		33 (57.9%)
Abnormalities on HRCT thorax	Ground glass opacities	33 (57.8%)
	Traction Bronchiectasis	25 (43.8%)
	Honeycombing	16 (28.07%)
Background immunosuppressants used	Mycophenolate mofetil	36 (63.2%)
	Hydroxychloroquine	10 (17.5%)
	Cyclophosphamide	7 (12.3%)
	Methotrexate	4 (7.02%)
Initiated on antifibrotics	Nintedanib	17 (29.8%)

**Table 3:** Comparison of patient profiles based on treatment groups SSc: Systemic sclerosis; ILD: interstitial lung disease; SD: standard deviation; NSIP: nonspecific interstitial pneumonia; FVC: forced vital capacity; TLC: total lung capacity; RV: residual volume; DLCO: diffusing capacity for carbon monoxide; 6MWD: six-minute walk distance; KBILD: King’s Brief Interstitial Lung Disease.

Characteristics	SSc ILD on immunosuppression only (n=40)	SSc ILD on immunosuppression and nintedanib (n=17)	p-value
Mean age in years ± SD	39.4 ± 11.2	37.9 ± 11.4	0.6
Anti-Scl-70 positive, n (%)	24 (60%)	9 (52.9%)	0.7
NSIP pattern, n (%)	32 (80%)	11 (64.7%)	0.3
FVC (% predicted) ± SD	48.2 ± 20.8	42.5 ± 17.5	0.3
TLC (% predicted) ± SD	67.9 ± 20.2	56.4 ± 19.1	0.05
RV/TLC (% predicted) ± SD	184.3 ± 121.4	152.08 ± 40.4	0.29
DLCO (% predicted) ± SD	50.5 ± 15.5	36.3 ± 11.5	0.001
6MWD (meters) ± SD	365.8 ± 86.09	347.3 ± 69.03	0.43
KBILD	66.8 ± 8.3	57.05 ± 12.6	0.001

## Discussion

Lung involvement in SSc occurs in nearly one-third of patients. However, clinically significant involvement occurs in 20% of patients with dcSSc and 12% of patients with limited cutaneous SSc [14]. A systematic review and meta-analysis found that the pooled prevalence of ILD among patients with SSc was 47% [15]. In our study, 26% of patients had clinically significant ILD. This variation in ILD prevalence may be due to a heterogeneous population and the detection of ILD at later stages.

We enrolled 57 patients after excluding those unable to perform lung function tests. Of these, 92.9% (n=53) were female patients, similar to a large US cohort of SSc patients, where 86% were female [16]. Female patients are more commonly affected than males, with an annual-adjusted incidence rate of 1.8 per 100,000 in women compared to 0.7 per 100,000 in men [17]. The age at diagnosis of SSc ranges from 33.5 to 55.8 years, with women typically diagnosed at a younger age than men (women: 49.2 ± 15.7 years; men: 58.9 ± 13.5 years) [18]. In our cohort, the mean age was 39 years, and the majority were women.

A study by Chan et al. reported mean predicted FVC, predicted DLCO, and 6MWD in SSc-ILD patients as 79 ± 21%, 57 ± 19%, and 395 ± 122 meters, respectively [19]. In our study, the mean predicted FVC, predicted DLCO, and 6MWD were 46.5 ± 19.9%, 46.2 ± 15.7%, and 360 ± 81.2 meters, respectively. These findings suggest that our patients had poorer lung function and lower exercise capacity at enrollment, indicating late presentation and a higher risk of poor outcomes. Radiologically, we found that the nonspecific interstitial pneumonia pattern was the most common ILD pattern, consistent with other studies [19]. ILD is more common in patients with dcSSc and anti-Scl-70 autoantibodies, similar to our findings [20]. A study by Ghuman et al. identified male gender and elevated C-reactive protein (>6 mg/ml) as prognostic and predictive markers of SSc-ILD outcomes [21].

Therapeutic options for SSc-ILD include cyclophosphamide (oral and intravenous), mycophenolate mofetil (MMF), tocilizumab, antifibrotics, and stem cell transplantation [22]. The Scleroderma Lung Study (SLS) 1 trial was the first to study the effect of cyclophosphamide in ILD, followed by the Fibrosing Alveolitis in Scleroderma Trial [23,24]. The SLSII trial compared cyclophosphamide and MMF [25]. The Safety and Efficacy of Nintedanib in Systemic Sclerosis (SENSCIS) trial evaluated nintedanib in SSc-ILD [7]. The primary endpoints in these studies included changes in FVC and DLCO over 12 to 24 months. One year of oral cyclophosphamide and two years of MMF showed similar efficacy [25]. The combination of MMF and nintedanib resulted in a slower decline in FVC compared to MMF or nintedanib alone, as observed in the SENSCIS trial [7].

In our study, most patients were treated with MMF and low-dose steroids. Nintedanib was initiated in patients with more than 10% lung involvement in HRCT. These patients had lower TLC, lower DLCO, and poorer KBILD scores than those on immunosuppressants alone. This suggests that high fibrosis scores negatively impact lung function and quality of life. Therefore, early initiation of antifibrotics in these patients may halt disease progression. All drugs were reasonably well-tolerated, with no patients discontinuing due to intolerance. Highland et al. conducted a subgroup analysis of the SENSCIS trial and found that the adverse event profile of nintedanib was similar regardless of concurrent MMF use, and the combination was well-tolerated, consistent with our findings [26].

Limitations

Our study had several limitations. It is a single-center study, which may introduce bias. Long-term follow-up data are still pending. Additionally, the safety and tolerability of another antifibrotic, pirfenidone, in combination with immunosuppressants have not been studied. Further large-scale, multicenter, randomized, placebo-controlled studies are needed to refine the treatment of SSc-ILD patients.

## Conclusions

SSc-ILD is relatively common among patients with SSc. All SSc patients experiencing dyspnea should undergo HRCT of the thorax as part of the ILD evaluation. Our study found that women are the predominant group and are likely to present at an earlier age. The presence of anti-topoisomerase antibodies is a significant risk factor for developing ILD. Early diagnosis is essential to preserve lung function and quality of life, as all known drugs can reduce the rate of decline in FVC. Cyclophosphamide and MMF are well-tolerated with no major adverse effects. The combination of MMF and nintedanib can be safely used in patients with SSc-ILD. Our results underscore the importance of early diagnosis and intervention in managing SSc-ILD to slow disease progression and preserve lung function. The high prevalence of ILD among patients with dcSSc and anti-topoisomerase antibodies highlights the need for vigilant screening in these high-risk groups. The tolerability of MMF, cyclophosphamide, and nintedanib suggests that these medications can be safely incorporated into treatment regimens, offering a potential strategy to improve patient outcomes. Further research should explore long-term outcomes and the efficacy of combining different antifibrotics and immunosuppressants to optimize treatment protocols for SSc-ILD.
